# Ovarian Gynandroblastoma with a Juvenile Granulosa Cell Tumor Component in a Postmenopausal Woman

**DOI:** 10.3390/diagnostics10080537

**Published:** 2020-07-30

**Authors:** Soohyun Hwang, Byoung-Gie Kim, Sang Yong Song, Hyun-Soo Kim

**Affiliations:** 1 Department of Pathology and Translational Genomics, Samsung Medical Center, Sungkyunkwan University School of Medicine, Seoul 06351, Korea; soohyun.hwang@samsung.com (S.H.); yoda.song@samsung.com (S.Y.S.); 2 Department of Obstetrics and Gynecology, Samsung Medical Center, Sungkyunkwan University School of Medicine, Seoul 06351, Korea; bgkim@skku.edu

**Keywords:** ovary, gynandroblastoma, juvenile granulosa cell tumor, postmenopausal woman

## Abstract

Ovarian gynandroblastoma (GAB) is an extremely rare sex cord-stromal tumor showing morphological evidence of both female (granulosa cell tumor) and male (Sertoli–Leydig cell tumor (SLCT)) components. Almost all GAB cases have been reported in children, adolescents, or women of reproductive age, and most of them typically have adult granulosa cell tumors as the female component. In contrast, GAB with a juvenile granulosa cell tumor (JGCT) component is a very rare condition; to the best of our knowledge, only one case of GAB with JGCT in a postmenopausal woman has been reported. In this report, we present an extremely rare case of ovarian GAB with JGCT in an elderly patient. A 65-year-old woman presented with an abdominal mass. Abdominopelvic magnetic resonance imaging revealed a large multiseptated cystic mass measuring 20 cm. No peritoneal seeding, lymph node enlargement, or hematogenous metastasis was identified. Laboratory test showed a slight elevation of serum CA 125 level (37.1 U/mL). Based on the preoperative clinical impression of ovarian cancer, she underwent a total hysterectomy with bilateral salpingo-oophorectomy. Grossly, the ovarian mass had a smooth and glistening surface without excrescences. The cut sections showed yellow-to-tan solid areas with foci of necrosis, myxoid degeneration, and hemorrhage, as well as multilocular cystic cavities filled with serosanguinous fluid. Histologically, the female component was characterized by JGCT displaying nodular growth patterns with follicle-like structures of various shapes and sizes. Most of the microcysts contained eosinophilic or basophilic secretions. The JGCT cells had indistinct cell borders, an abundant eosinophilic cytoplasm, and round-to-oval hyperchromatic nuclei with many mitotic figures. The SLCT component consisted predominantly of intermediately differentiated Sertoli cells forming lobulated solid nodules. They were arranged in cords, solid tubules, or nests, and possessed oval-to-spindle-shaped darkly stained nuclei and scant cytoplasm. In several foci, well-formed Sertoli cell tubules were loosely aggregated within areas of moderately differentiated SLCT. In summary, we described GAB in a postmenopausal woman with JGCT and SLCT as the female and male components, respectively. This is the second case of GAB with JGCT occurring in an elderly patient. Our findings can help pathologists and clinicians make accurate histological diagnoses of GAB with a JGCT component and plan an adequate treatment strategy for this rare tumor.

**Figure 1:**
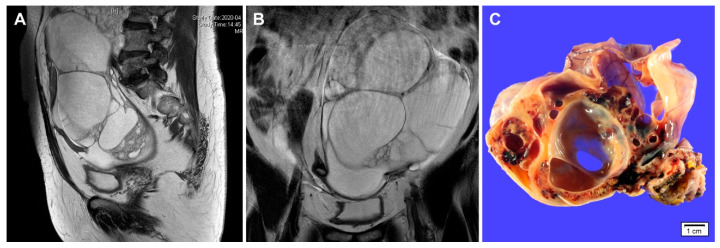
Imaging and gross findings of gynandroblastoma. (**A**,**B**) a 65-year-old woman presented with an abdominal mass. Abdominopelvic magnetic resonance imaging reveals a 20 cm multiseptated cystic mass arising from the left ovary. No evidence of abdominopelvic peritoneal seeding, lymph node enlargement, or hematogenous metastasis is identified. Preoperative laboratory test showed a slight elevation of serum CA 125 level (37.1 U/mL). The serum CA 19-9 level was within normal range (32.1 U/mL). (**C**) Grossly, the cut section of the ovarian tumor shows variable-sized, thin-walled cystic spaces. Solid areas show yellow-to-tan tumor tissue with hemorrhagic spots. The inner surface of cystic lesions is relatively smooth and glistening.

**Figure 2:**
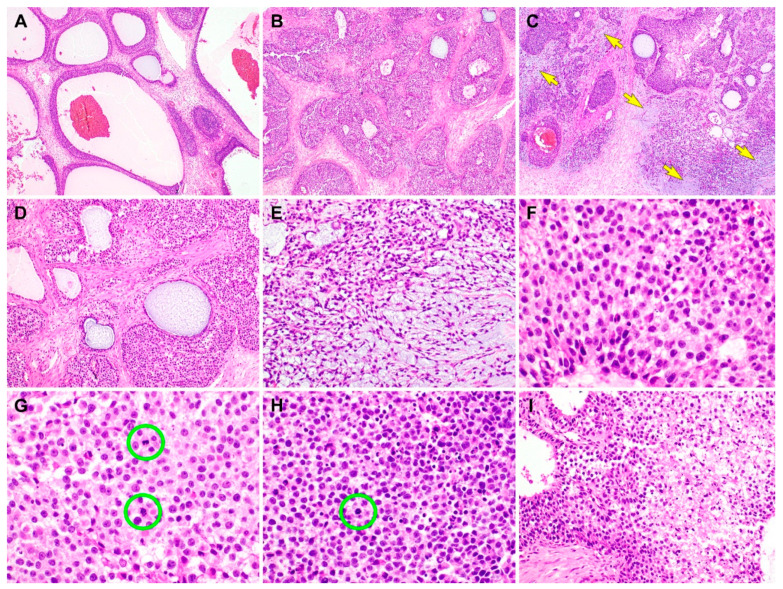
Histological findings of the juvenile granulosa cell tumor (JGCT) component of gynandroblastoma. (**A**) The tumor has many follicles that vary in size and shape [1]. The thickness of the cells lining the follicles also varies. (**B**) The tumor cells form variable-sized solid nodules punctuated by small microcysts. The intervening stroma is fibrotic or edematous. (**C**) Several foci show basophilic matrices (yellow arrows) that are located adjacent to or merge with the solid cellular nodules. (**D**) The follicles contain secretions that are eosinophilic, though they are more often basophilic. (**E**) In the background of the basophilic, myxoid stroma, the bipolar or stellate-shaped cells proliferate diffusely. (**F**) On high-power magnification, the JGCT cells typically possess an abundant eosinophilic or a pale amphophilic cytoplasm. Their nuclei are round to oval and lack intranuclear grooves, one of the characteristic features of adult granulosa cell tumor. Note mild-to-moderate nuclear pleomorphism, uniform hyperchromasia, and occasional conspicuous nucleoli. (**G**) Mitotic figures (green circles) are frequently identified, and they number up to 8 per 10 high-power fields. (**H**) In addition to the mitotic figures (green circle), there are some microscopic foci showing karyorrhectic debris. (**I**) In a few foci, areas of coagulative tumor cell necrosis are noted at the central portions of solid cellular nodules. The hematoxylin and eosin stain were used (**A**–**I**). The original magnifications were 40× in **A**–**C**, 100× in **D**, 200× in **E**, 400× in **F**–**H**, and 200× in **I**.

**Figure 3:**
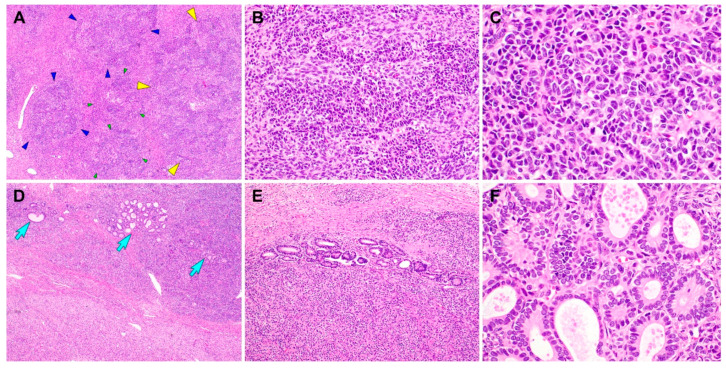
Histological findings of the Sertoli–Leydig cell tumor (SLCT) component of gynandroblastoma. (**A**) Moderately differentiated SLCT shows a multilobulated growth pattern on low-power magnification, with areas of alternating hypo- and hypercellularity. Each of the variable-sized lobules is outlined by green, blue, or yellow arrowheads. (**B**) The proliferating Sertoli cells form cords, solid tubules, and nests. (**C**) On high-power magnification, the Sertoli cells exhibit oval-to-spindle-shaped, hyperchromatic nuclei with mild atypia and a scant cytoplasm. (**D**,**E**) In several foci, loosely aggregated or individually scattered Sertoli tubules with open or compressed lumina (blue arrows) are readily identified. (**F**) The lining cells are low columnar-to-cuboidal cells and lack significant nuclear atypia. No evidence of poorly differentiated SLCT, a heterologous component, or coagulative tumor cell necrosis is observed. The hematoxylin and eosin stain were used (**A**–**F**). The original magnifications were 40× in **A**, 200× in **B**, 400× in **C**, 40× in **D**, 100× in **E**, and 400× in **F**.

**Figure 4:**
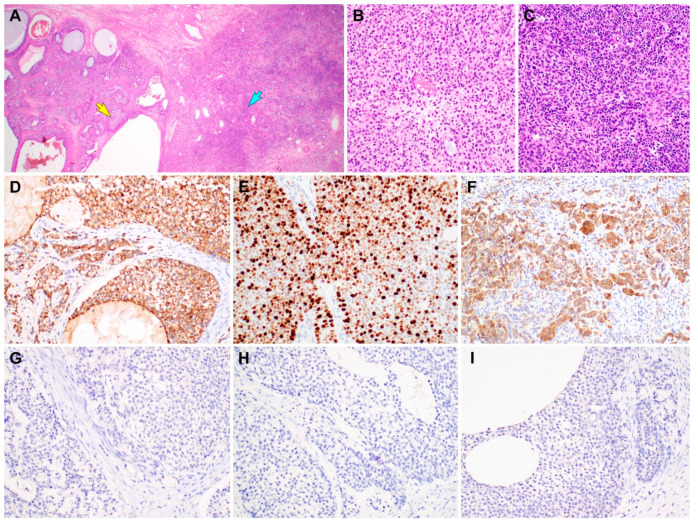
Immunostaining results of gynandroblastoma (GAB). (**A**) Both the juvenile granulosa cell tumor (JGCT; left half) and Sertoli-Leydig cell tumor (SLCT; right half) components are seen. (**B**) High-power magnification of the area indicated by a yellow arrow in image A reveals solid sheets of JGCT cells possessing an abundant eosinophilic cytoplasm. (**C**) High-power magnification of the area indicated by a blue arrow in image A reveals a diffuse proliferation of darkly stained Sertoli cells forming vague cords and nests. (**D**) Inhibin-α immunostaining uniformly highlights the JGCT cells. (**E**) The JGCT component exhibits high Ki-67 labeling index (approximately 70%). (**F**) The SLCT component also shows uniform cytoplasmic inhibin-α immunoreactivity. (**G**–**I**) In contrast, the JGCT and SLCT cells are negative for (**G**) epithelial membrane antigen, (**H**) pan-cytokeratin, and (**I**) paired box 8. Based on the morphological evidence of both the female and male components, lack of epithelial and Mullerian lineage markers, and inhibin-α immunoreactivity [2,3,4,5,6,7,8], the final histological diagnosis of ovarian GAB with a JGCT component was established. She did not receive any further treatment such as postoperative chemotherapy or radiation therapy. Two months after surgery, she is well without evidence of recurrent disease or distant metastasis. To the best of our knowledge, there has been only a single case of GAB with JGCT diagnosed in a postmenopausal woman [9,10,11]. This is the second case of GAB with JGCT in an elderly woman who had been in post-menopause for 15 years. The stains used were hematoxylin and eosin stain in **A**–**C** and polymer method in **D**–**I**. The original magnifications were 12.5× in **A** and 200× in **B**–**I**.

## Abbreviations

GAB	Gynandroblastoma
JGCT	Juvenile granulosa cell tumor
SLCT	Sertoli-Leydig cell tumor
